# An Unusual Case of Concurrent Herpes Simplex Virus Type 1 and Cytomegalovirus Encephalitis Complicated by Primary Central Nervous System Lymphoma as an Initial Presentation of Acquired Immunodeficiency Syndrome

**DOI:** 10.7759/cureus.19601

**Published:** 2021-11-15

**Authors:** Dawood Findakly

**Affiliations:** 1 Hematology and Medical Oncology, Louisiana State University Health Sciences Center, Shreveport, USA

**Keywords:** primary central nervous system lymphoma, aids, brain biopsy, chemotherapy, hiv-directed therapy

## Abstract

Primary central nervous system lymphoma (PCNSL) is an uncommon brain tumor with a puzzling diagnosis. It has an incidence of seven cases per 100,000,000 people in the United States, which is further lower in immunocompromised patients. Cytomegalovirus (CMV) is a common cause of various malignancies, notably Burkitt’s lymphoma, nasopharyngeal carcinoma, Hodgkin’s lymphomas, and non-Hodgkin’s lymphomas (NHL) including PCNSL. Cases with PCNSL can vary in presentation with either focal or non-focal manifestations progressively worsening over a period that could last up to a few months. In this report, we discuss the case of a 39-year-old woman with a past medical history of bipolar disorder who presented with PCNSL as the initial presentation of acquired immunodeficiency syndrome (AIDS). This case report emphasizes the importance of a multidisciplinary team (MDT) approach for the interpretation as well as for correlating the laboratory and imaging results with clinical findings given the challenging diagnosis, to choose an appropriate management approach that is tailored to the patient's presentation.

## Introduction

Herpes simplex virus type 1 (HSV-1) encephalitis is an uncommon condition. Its co-infection with cytomegalovirus (CMV) is mostly reported in immunocompromised adults, especially those with human immunodeficiency virus (HIV) [1]. Primary central nervous system lymphoma (PCNSL) is a rare and highly aggressive type of extranodal non-Hodgkin's lymphoma (NHL), with the highest incidence reported in immunocompromised patients. PCNSL affects the central nervous system (CNS) including the brain parenchyma, eyes, spinal cord, and spinal cord without systemic involvement [2]. PCNSL has a favorable response to chemotherapy and radiation therapy in contrast with other brain tumors, but it is associated with mediocre survival rates when compared with lymphomas outside the CNS [3]. In this case report, we present a middle-aged woman with PCNSL as the initial presentation of acquired immunodeficiency syndrome (AIDS).

## Case presentation

A 39-year-old woman with a past medical history of bipolar disorder was brought to the emergency department (ED) with the chief complaint of oral thrush. Upon initial evaluation, the patient could not specify when the thrush had begun but denied dysphagia, odynophagia, excess secretions, previous history of an immunocompromised state, or HIV infection. Meanwhile, she could not provide any explicit history; she was awake but only alert and oriented to self and date of birth. Her family stated that she had not been caring for herself before her presentation. In the ED, her vitals were significant for blood pressure of 110/71 mmHg, heart rate of 63 bpm, respiratory rate of 19 bpm, a temperature of 37.1 °C, and body mass index of 16.57 kg/m^2^. Labs were significant for white blood cell (WBC) count of 4.2 × 10^3^/µL, relative lymphocytes of 11%, sodium of 132 mEq/L, alanine aminotransferase (ALT) of 67 U/L, and aspartate aminotransferase (AST) of 81 U/L. Her HIV 1 antibody test came back positive, and CT of the head without contrast showed mild generalized parenchymal volume loss that was greater than expected for the patient's age. Upon physical examination, the patient appeared to be cachectic with temporal wasting; she was alert and oriented to only person and place, but not time. Interestingly, eye examination showed involuntarily, random, non-purposeful bilateral eye movements consistent with opsoclonus. The oral mucosa examination revealed minimally scrapable white plaques on the tongue, gums, and buccal mucosa bilaterally with multiple dental caries. She was noticed to have severe muscle wasting more prominent to the temporalis, pectoralis, interosseous, and gastrocnemius muscles with moderate subcutaneous fat loss noted in the orbital region. Cardiac, pulmonary, and abdominal examinations were unremarkable.

**Figure 1:**
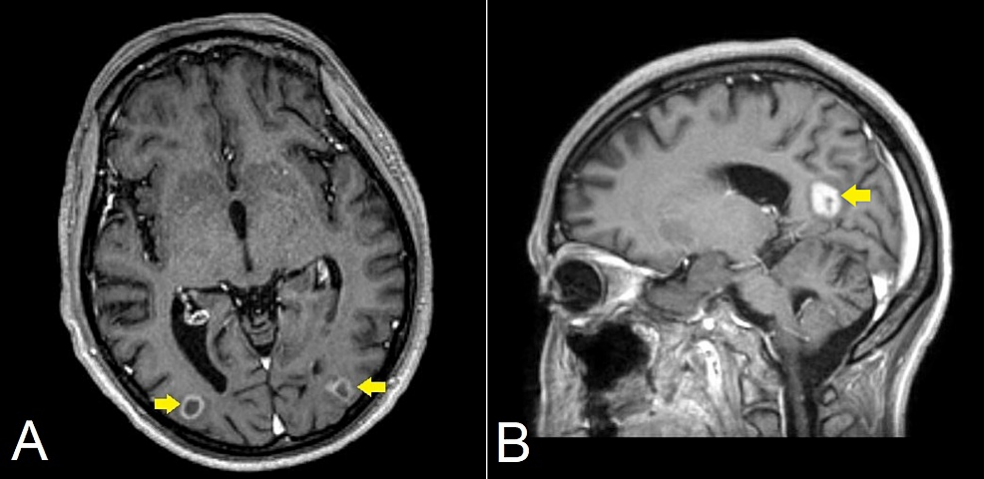
Axial (A) and sagittal (B) T1-weighed contrast-enhanced MRI images in a patient with PCNSL The images show multiple contrast-enhanced ring-enhancing lesions (yellow arrows) within the brain parenchyma with adjacent vasogenic edema MRI: magnetic resonance imaging; PCNSL: primary central nervous system lymphoma

A lumbar puncture was performed, and the cerebrospinal fluid (CSF) was clear with an opening pressure of 15 cmH_2_O. In the meantime, the patient was started empirically on vancomycin, ceftriaxone, acyclovir, and atovaquone given lymphopenia, and fluconazole given oral thrush. CSF glucose was 34 mg/dl, protein was 147 mg/dl, and WBC was 10/mm^3^. CSF workup showed HSV-1 and CMV infection, and CSF culture with gram stain showed no organisms. HIV viral load was 728,190 copies/mm^3^ and CD4 count was 9 cells/mm^3^. The infectious disease (ID) team recommended discontinuing vancomycin and ceftriaxone given negative CSF cultures. In the meantime, ophthalmology evaluated the patient and reported no evidence of CMV retinitis or retinal lymphoma. MRI revealed findings of ventriculitis and multiple parenchymal ring-enhancing lesions with surrounding vasogenic edema (Figure 1). Given the patient's HIV status, the findings raised suspicion for toxoplasmosis vs. PCNSL. Toxoplasmosis was ruled out as serologies were negative for toxoplasmosis, and hence neurosurgery was consulted for a brain lesion biopsy for suspected lymphoma.

**Figure 2:**
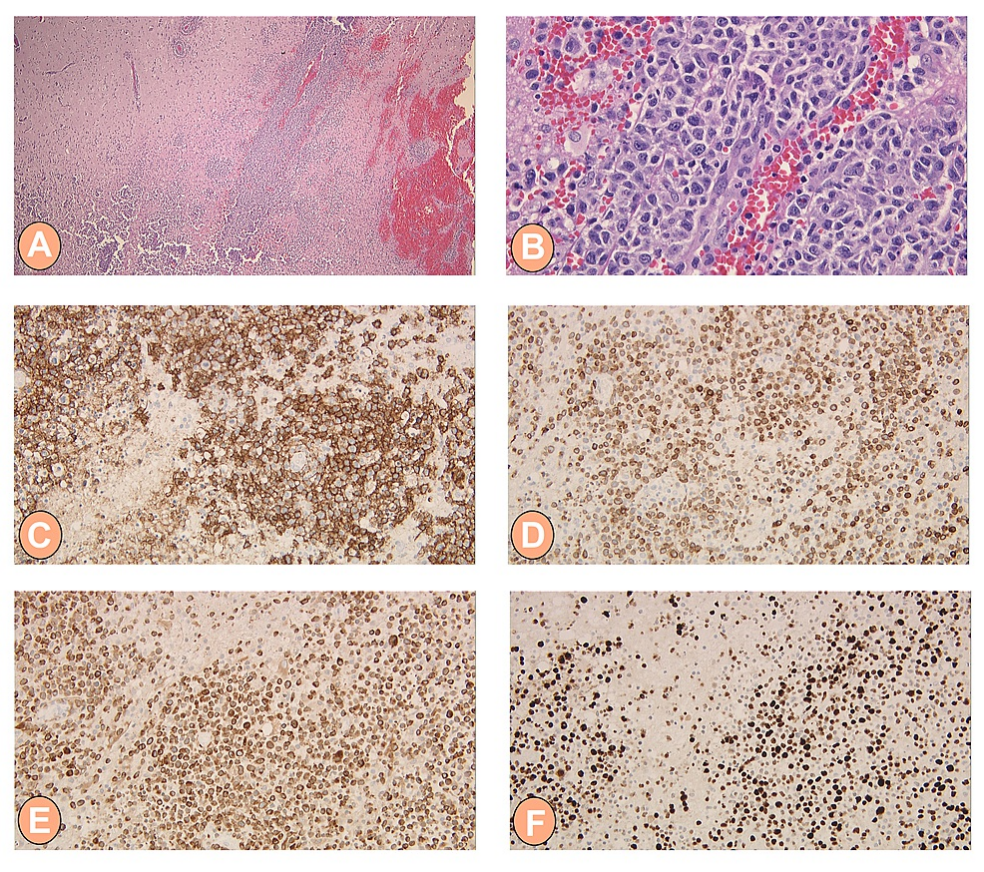
Histologic features of PCNSL H&E staining of the biopsy sample demonstrating the perivascular and cortical parenchymal infiltration pattern of PCNSL (A) at 2x magnification, and (B) at 40x magnification showing large and pleomorphic cells with irregular nuclear contours, granular to vesicular chromatin, and prominent nucleoli. Moreover, IHC stains on tumor cells (at 20x magnification) were positive for (C) CD20, (D) CD79a, (E) BCL2, and (F) Ki-67 with 60-65% positivity PCNSL: primary central nervous system lymphoma; H&E: hematoxylin and eosin; IHC: immunohistochemistry; CD: cluster of differentiation; BCL2: B-cell lymphoma 2; Ki-67: an antigen and marker of proliferation

Further imaging with a CT scan of the chest, abdomen, and pelvis reported no metastatic disease; no site for primary lymphoma was found, and there was no evidence of systemic lymphadenopathy or splenomegaly. Baseline cardiac function was obtained through an echocardiogram. Lateral occipital craniotomy excisional biopsy was performed and brain biopsy reported tissue involvement by diffuse large B-cell lymphoma (DLBCL) with perivascular and cortical parenchymal infiltration by cells positive for CD20, CD79a, and BCL2 with Ki-67 showing 60-65% positivity while negative for CD3, CD5, CD10, CD138, and Cyclin D1. Few scattered cells (<10%) showed staining with BCL6 and MUM-1 stain was positive in large lymphocytes (Figure 2).

Following the confirmation of the diagnosis, a multidisciplinary team (MDT) comprising hematology-oncology and ID teams arranged to start highly active antiretroviral therapy (HAART) in conjunction with chemotherapy with high-dose methotrexate (HD-MTX) therapy with an anticipated duration of therapy on the order of six cycles recommended followed by consolidation therapy. Accordingly, the patient received cycle 1 HD-MTX at 3.5g/m^2^ with leucovorin rescue, urinary alkalinization, and granulocyte-colony stimulating factor (GCSF) support. Given highly emetogenic chemotherapy with prophylactic fosaprepitant, palonosetron, dex, and olanzapine were warranted. The patient tolerated the first cycle of chemotherapy well, and her mentation improved significantly. Repeat CSF analysis on day 14 of therapy indicated the eradication of HSV-1 and CMV infections. While remaining in the hospital, the patient received the second chemotherapy protocol. The patient continued to improve clinically (methotrexate level of <0.1 µM), and she was discharged home with hematology-oncology follow-up in the outpatient setting.

## Discussion

Patients with HIV infection are at a greater risk of developing a variety of cancers, with a more aggressive clinical course compared to cancers in non-HIV-infected patients [4]. Their immunocompromised state directly results in a larger incidence of cancers in patients with HIV infection, similar to the case with transplant-recipient populations [5].

AIDS-defining malignancies include Kaposi's sarcoma, NHL, and invasive cervical carcinoma [4]. PCNSL is a highly aggressive type of B-cell NHL that is typically confined to the CNS, including the brain, spine, CSF, and eyes. It represents less than 5% of intracranial tumors and less than 15% of NHLs in HIV-infected patients with the most common histology being DLBCL [6,7]. Most PCNSLs occur in the immunocompetent hosts with a higher incidence in immunocompromised patients, including patients on immunosuppressive medications, those with HIV infection, or those who undergo organ transplantation. It has a variable presentation based on the area of the CNS involved, whether brain parenchyma or leptomeninges [8].

CSF evaluation for the disease, an MRI of the entire spinal axis for tumor characterization, and a slit-lamp ophthalmologic examination are vital to rule out vitreoretinal involvement as it could harbor lymphoma and be a site for recurrence [9]. Moreover, before starting chemotherapy, cardiac evaluation and a central venous access line are needed in anticipation of chemotherapy. Baseline renal function is essential and it is very important to avoid nephrotoxic agents while treating patients with this condition [10].

There has been significant progress in the treatment of PCNSL over the past few decades. According to the current guidelines, HD-MTX is the backbone of PCNSL treatment [11]. It is given in a 14-day cycle, requiring about five days of hospitalization each time, to be administered in six cycles. The reason for the duration of hospitalization is usually to ensure that the HD-MTX level is safe for discharge. This requirement could affect the disposition and placement plans and increase the burden on patients receiving chemotherapy.

AIDS-related PCNSL can be effectively treated by the initiation of HD-MTX in conjunction with HIV-directed antiretroviral therapy, leading to long-term survival with decreased recurrences [12]. The clinical course and survival outcomes are tightly linked to patient-specific factors, and circumstances related to management, including delayed diagnosis, and therapy [13]. Therefore, it is important to adhere to the guidelines regarding the management by starting HAART concurrently with HD-MTX and following an MDT approach after addressing the benefits and risks of the initiation of therapy with patients involved in the decision-making process.

## Conclusions

A high index of suspicion for the early recognition of the PCNSL is crucial given its variable manifestations and potentially challenging clinical syndromes. In our case, the HSV-1 and CMV infections complicated PCNSL, revealing a new AIDS diagnosis. Hence, an MDT approach comprising neurosurgery, ID, and hematology-oncology is essential for its prompt management and to improve outcomes. This case report further highlights the effectiveness of a combined modality in the treatment of PCNSL and its comorbid conditions.
